# Introducing a Novel Method of Patella Tendon Defunctioning using Suture Anchors after a Tibial Tuberosity Avulsion Repair: Report of Three Cases

**DOI:** 10.5704/MOJ.2107.023

**Published:** 2021-07

**Authors:** EJY Cheong, LTJ Tan

**Affiliations:** Department of Sports Orthopaedics, Tan Tock Seng Hospital, Singapore

**Keywords:** sports, tibial tuberosity avulsion injury, defunctioning patella tendon, suture anchors, defunctioning using suture anchors

## Abstract

Tibial tuberosity avulsion injuries are rare and result from direct trauma to the tibial tuberosity or forceful and repetitive contraction of the quadriceps muscles. In this case series, we describe a novel method of defunctioning the patella tendon using a suture anchor after a tibial tuberosity avulsion fracture repair was performed. We present three consecutive patients with tibial tuberosity avulsion fractures who were treated by the same surgeon using the technique described. Pre and post-operative range of motion of the knee joint were then reviewed retrospectively. All patients achieved pre-injury range of motion within three months post-operatively. In conclusion, defunctioning the patellar tendon with a suture anchor is a reliable and reproducible technique. The new technique yielded excellent functional outcomes which allowed patients to regain their pre-injury range of motion and the strength of the construct allows early range of motion.

## Introduction

Tibial tuberosity avulsion fractures (TTAF) are rare and leaving them untreated could lead to significant functional limitations. Surgery remains the mainstay of treatment in conjunction with bracing and activity modification. Current surgeries aim to restore mobility by reducing and securing the avulsed fragment and, often, a wire loop is used to defunction the patella tendon^1^. However, there are limitations to current methods. Cerclage wires require removal ten weeks after surgery due to potential wire breakage which would lead to tissue injury^2^. Consequently, the need for an additional procedure increases infection risks, soft tissue damage and healthcare cost^3^.

In this case series, we describe three cases in which suture anchors were used to defunction the patella tendon after a TTAF repair instead of wires. Suture anchors facilitate the healing process by optimising the link between bone and soft tissue and providing physiological off-loading on the repair site^4^. Currently, there is a case report published^5^ describing the same technique of utilising suture anchors after a TTAF repair and it showed equivalent outcome to that of conventional techniques.

**Fig. 1:**
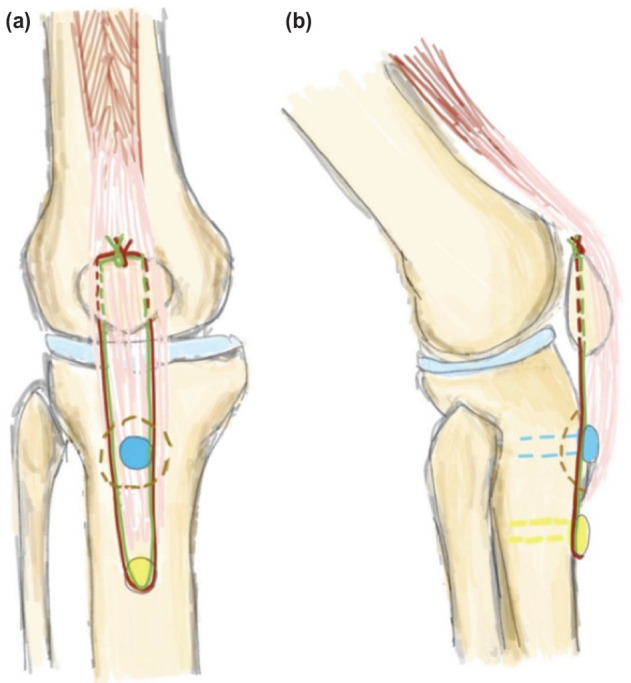
(a) Anterior (b) and lateral view of right knee.

**Fig. 2:**
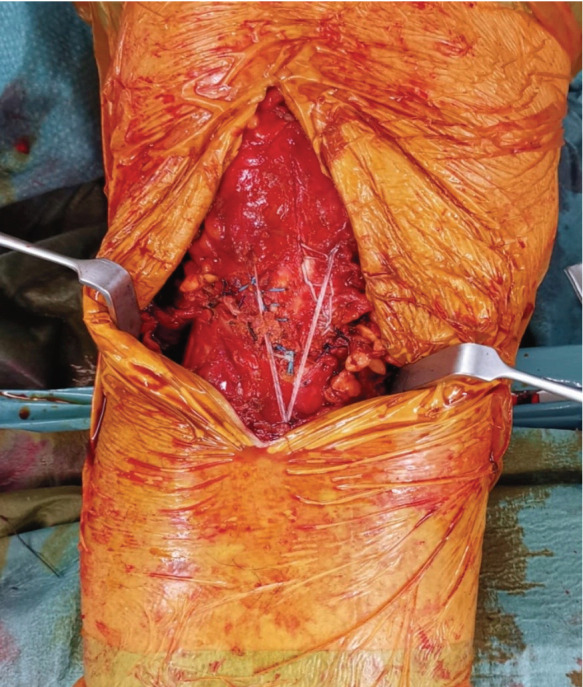
Post-operative picture after application of new defunctioning technique.

We describe the technique as illustrated in Fig. 1a and b. Under anaesthesia, a midline incision is made over the course of the patella tendon and the fracture is identified. The fracture edges are freshened until bleeding bone is visible, then reduced and fixed with a screw (blue pin). Depending on the size of our patient, either a 4.5mm or 5.5mm suture anchor (yellow pin) is secured 2cm inferior to the tibial tuberosity. Two 2.4mm vertical drill holes are created in the patella using a Beath pin and the sutures are threaded through and knotted securely above the superior border of the patella (red and green lines). The completed repair and defunctioning construct is depicted by an intra-operative image in (Fig. 2). The repair site is then irrigated copiously and tissue is closed in layers and bandaged. The knee is secured with a brace locked at 0°.

## Case Report

### Case 1

A 53-year-old Chinese gentleman presented with left knee pain after sustaining direct trauma to his left knee after slipping on his way to work. Radiography revealed an avulsion injury at the superio-anterior aspect of the left tibia.

### Case 2

**Fig. 3:**
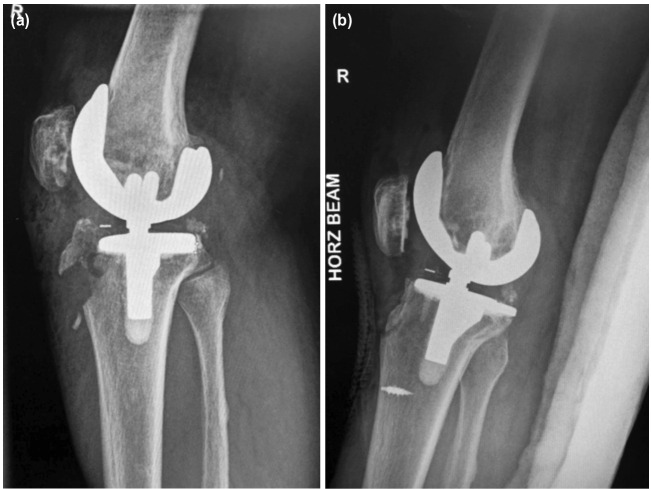
(a) Pre-operative, (b) post-operative radiograph lateral view of right knee (metallic anchor suture used here).

A 74-year-old Chinese lady with a history of total knee replacement (TKR) developed pain and inability to weight bear on her right lower limb after she fell and landed directly on her right knee. Her pre-injury range of motion (ROM) was noted to be 0° to 90° during routine follow-up for her TKR. Pre-operative radiographs revealed a TKR in-situ and an open right tibial tuberosity fracture as shown in Fig. 3a. Suture anchors were used to fix the fracture as well as to defunction the patella tendon. Her three month postoperative radiograph is depicted in Fig. 3b.

### Case 3

A 56-year-old Malay lady with history of osteopenia and transosseous repair of distal patella pole fracture slipped and sustained direct trauma to left knee. Her pre-injury ROM was 0° to 65° after the initial patella fracture. Radiography showed left tibial tuberosity avulsion injury and knee effusion.

The new technique yielded excellent results for all three patients. Table I provides a summary of the demographics, fixation methods and functional outcomes of our patients. At three and six months post-operative, all three patients achieved pre-operative ROM and a normal gait with no significant patella mal-tracking. There were no complications post-operatively and all incisions healed after three months. All three patients made good recovery and were satisfied with their functional outcomes.

**Table I:** A summary of our results

	Age (years)	Gender	Side of Knee	Pre-op ROM (degrees)	Three Months Post-op ROM (degrees)	Six months Post-op ROM (degrees)	Fixation Method
Case 1	53	Male	Left	Not Documented	0-150	0-150	Cancellous Screw With Washer
Case 2	74	Female	Right	0-90	0-90	0-90	Suture Anchor
Case 3	56	Female	Left	0-65	0-65	0-80	Cancellous Screw With Washer

## Discussion

Surgery remains the mainstay of treatment for TTAFs to properly restore the function of the affected limb. Following the fixation, a period of three to six months is required for adequate bone to bone union to be achieved. During this time, the repair should be protected to allow for earlier ROM. This has traditionally been done with a defunctioning cerclage wire, but this necessitates a second surgery to remove the wires later as previously discussed.

The points of fixation of our method are distal to the fracture site and proximally above the patella. This allows us to completely bypass the fracture site and the entire patella tendon, serving several purposes. Firstly, it reduces stress on the entire patella tendon by reducing the tensile pull on the fracture site. Secondly, the placement of the anchor in strong cortical bone ensures that the strongest fixation points are selected. Lastly, by spacing the tunnels apart, there will be an adequate bone bridge between the two transosseous tunnels, and hence can safely be used in elderly patients with osteopenic bones.

Ultimately, the surgeon needs to be familiar with what suture anchors are available to them. Factors which can affect the construct include the material of the anchor, the pull out strength and the pitch of the anchors as well as the strength of the sutures.

An independent case study of one patient published by Tai *et al*^5^ using a similar technique also yielded consistently positive outcomes. Despite the limited data thus far, the positive results observed in these two independent case studies increased our confidence in applying such technique to future patients.

In conclusion, defunctioning the patella tendon with a suture anchor is a reliable and reproducible technique. The new technique yielded excellent functional outcomes which allowed patients to regain their pre-injury ROM and the tensile strength of the construct allows for early ROM. We hope that this case series would increase awareness of this new defunctioning technique to orthopaedic surgeons globally.
